# Google Trends Analysis Reflecting Internet Users’ Interest in Selected Terms of Sexual and Reproductive Health in Ukraine

**DOI:** 10.3390/healthcare11111541

**Published:** 2023-05-25

**Authors:** Joanna Błajda, Anna Kucab, Aldona Miazga, Maciej Masłowski, Marta Kopańska, Anna Nowak, Edyta Barnaś

**Affiliations:** 1 Institute of Health Sciences, Medical College, University of Rzeszow, 35-959 Rzeszow, Poland; 2 Institute of Medical Sciences, Medical College, University of Rzeszow, 35-959 Rzeszow, Poland; 3 Center for Foreign Language Studies, University of Rzeszow, Aleja Rejtana 16c, 35-959 Rzeszow, Poland

**Keywords:** sexual health, reproductive health, war, Google Trends

## Abstract

Introduction: The war in Ukraine has had adverse impacts on all areas of life, including health-related issues. Limited access to medical care increases the need to look for alternative sources of medical information. Aim: To analyze trends of Internet users’ interest in sexual and reproductive health in Ukraine based on Google Trends. Materials and Methods: The retrospective study was based on the analysis of terms related to sexual and reproductive health searched by Ukrainian Internet users. The tool used was Google Trends. The period from 1 January 2021 to 1 January 2023 was analyzed. The time variability in search growths and peaks was assessed from the perspective of two time intervals (before the war and during the war) using the chi-square test. Results: Significant changes have been demonstrated in the interests of Internet users from Ukraine regarding selected issues of sexual and reproductive health during the ongoing war. Compared to the pre-war period, a marked increase was observed in active searches for terms such as “condoms” (*p* = 0.0081), “rape” (*p* = 0.0008), “syphilis” (*p* = 0.0136), “ovulation” (*p* = 0.0002) and “pregnancy test” (*p* = 0.0008). Conclusions: The conducted analysis clearly indicates an increased need for information regarding sexual and reproductive health among Ukrainian citizens during the ongoing armed conflict. The analysis of trends among Internet users interests can be a valuable source of knowledge for decision makers, including human rights organizations, regarding the scope and coordination of activities aimed at protecting the sexual and reproductive health of the inhabitants of Ukraine.

## 1. Introduction

Reproductive health is a component of sexual health. According to the WHO, it is a set of biological, emotional, intellectual and social aspects of sexual life, necessary for the positive development of human personality, communication and love. The definition given clearly classifies that sexuality should be based on respect, safety and freedom from discrimination [1]. Armed conflicts prevent the full realization of all the assumptions that are assigned to the defined terms. The war in Ukraine has resulted in a crisis in access to safe childbirth and neonatal care, contraceptives to prevent unwanted pregnancy, safe abortion, comprehensive sex education for youth, prevention and treatment of HIV and other sexually transmitted diseases, and protection of people of different sexual orientations and gender identity [2]. The global organization Sexual and Reproductive Health and Research emphasizes that attention should be paid to the impacts of conflicts and forced migration on the sexual and reproductive health of Ukrainian citizens [3].

Sexuality as well as contraception are no longer taboo topics; the key role in this matter is played by open access to information via the Internet, which is a popular source of knowledge about health. In the opinion of users, the Internet gives a great sense of freedom in searching for information [4]. Nearly three-quarters of Internet users look for health information on the Internet [5]. The role of the Internet during armed conflict can be of particular importance as it is frequently the only available source of information. For numerous Internet users, it is the first-choice source of information before seeking advice from a medical specialist [6]. A Research & Branding Group survey from February 2021 demonstrated that the Internet was, for the first time, the main source of information (51% preferred the Internet and 41% television) for Ukrainians [7].

According to the international ranking conducted in February 2022, the most popular Internet search engine in the world is Google (Google—73.95%, Bing—15.81%, Baidu—3.88%) [8]. An interesting Google service is the free tool Google Trends (GT), a service that provides daily insight into what the world is searching for on Google, showing the relative amount of search traffic for any query in any language depending on the time and region of queries directed to Google Search. The analysis of web search trends can be useful for advertisers, economists, scientists and all other people interested in knowing what Internet users are interested in [9,10,11].

The subsequent parts of the paper include the purpose of the work, the methodology of the research conducted and a list of the keywords analyzed. The results of the analysis of 22 keywords, including the peaks of interest in them both before and after 24 February 2022, were analyzed. The relevance of the results is compared with the literature in the discussion section, making arguments for the conclusions section.

### Aim

The aim of the study was to analyze trends of Internet users’ interest in sexual and reproductive health in Ukraine based on the Google Trends tool.

## 2. Material and Method

In this retrospective study, a selection of sexual and reproductive health keywords searched on the Internet were analyzed. The tool used was Google Trends, with the help of which we obtained data on the number, origin, dependence on time and main regions of queries directed to the Google search engine. The ranking of the obtained entries was sorted by language (all entries were entered in Ukrainian); the entries were also sorted by country (the territorial scope was selected—Ukraine).

The analyzed keywords were selected in a multi-stage process. Initially, keywords were chosen by brainstorming possible words that we thought were common in the fields. In addition, we checked what other GT researchers had analyzed on related topics to find out about any important topics or keywords that we might have missed. While performing the analysis in GT, we also paid attention to similar topics displayed in GT, which were a hint of what other terms were being searched by other users searching for terms similar to ours. Keywords for which the number of searches did not reach the minimum value threshold in the analyzed time were excluded. Table 1 is presented below.

The study was conducted between 2 and 5 January 2023. The period from 1 January 2021 to 1 January 2023 was analyzed. According to the Google Trends algorithm, search interest was quantified on a normalized scale of 0–100 GT, with the highest indicator assigned to 100. The study included, in detail, the growths and peaks of searches on a scale of 0–100 GT. Keywords for which the number of searches did not reach the minimum value threshold in the analyzed time were excluded. The time variability in the search was assessed from the perspective of two time intervals, i.e., before 24 February 2022, prior to the outbreak of the war, and after 24 February 2022, during the war. We used the goodness-of-fit chi-square test and checked whether the observed proportions differed from the expected values, assuming an even distribution of results (50% each before and after the war). This is otherwise known as the chi-2 concordance test. It was assessed whether the numbers in terms of interest in given terms were significantly different in these time intervals. The chi-square test was used assuming a significance level of *p* < 0.05.

## 3. Results

The conducted analysis showed that in the case of 15 out of 22 identified keywords, the peak of interest occurred during the war (after 24 February 2022)—Table 2.

**Figure 1:**
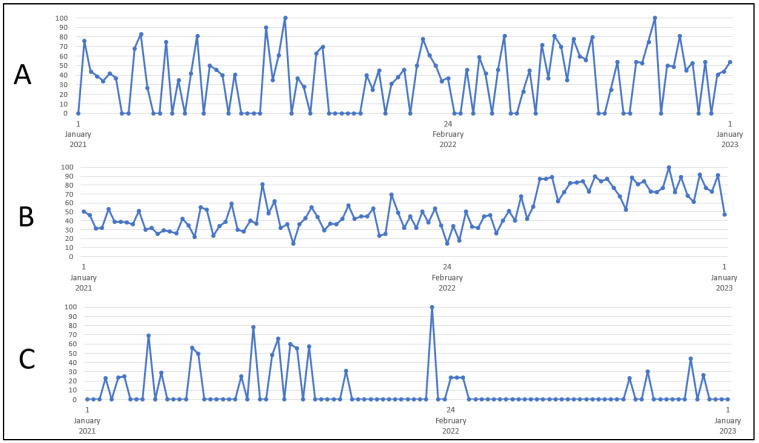
Internet users’ interest in the following keywords: (**A**) “contraception”, (**B**) “condoms”, (**C**) “intrauterine device”. Source: trends.google.com.

The greatest interest in the keyword “contraception” was recorded on 25–31 August 2021 and 16–22 October 2022 (100 on the GT scale)—Figure 1. The greatest interest in the term “emergency contraception” was recorded on 24–30 July 2022 (GT 100). The peak of interest in the term “morning-after pill” was recorded between 28 February and 6 March 2021. The research showed no significant difference in interest in the terms “contraception” (*p* = 1.0000), “emergency contraception” (*p* = 0.1349) and “morning-after pill” (*p* = 0.6658) during the war compared to the time before the conflict—Table 2.

The most frequently searched term in the field of contraception was “condoms”. The greatest interest in the term “condoms” was recorded between 30 October and 5 November 2022 (100 on the GT scale)—Figure 1. The analysis showed a significant increase in interest in the term “condoms” during the war compared to the time before the conflict (*p* = 0.0081)—Table 2.

The greatest interest in the term “contraceptive pills” was recorded between 20 November and 26 November 2022 (GT scale 100). The results did not show contrast in the interest in this term in the analyzed time periods (*p* = 0.4670)—Table 2.

The greatest interest in the term “intrauterine device” in the analyzed time period was recorded on 2–8 January 2022 (100 on the GT scale)—Figure 1. The analysis showed a significant decrease in interest in the term “intrauterine device” during the war compared to the time before the conflict (*p* < 0.0001). The lowest interest in the analyzed time period was recorded from 5 March 2022 to 30 July 2022 and from 17 September 2022 to 19 November 2022 (0 on the GT scale)—Table 2.

The greatest interest in the term “abortion” was recorded on 10–16 January 2021 (GT scale 100). No differences were demonstrated in the comparison of the interest in this term in the analyzed time periods (*p* = 0.3046)—Table 2.

The greatest interest in the keyword “sexual violence” was recorded during the war on 24–30 July 2022 (100 on the GT scale). There was no significant difference in the interest in this term in the analyzed time periods (*p* = 0.3815)—Table 2.

**Figure 2:**
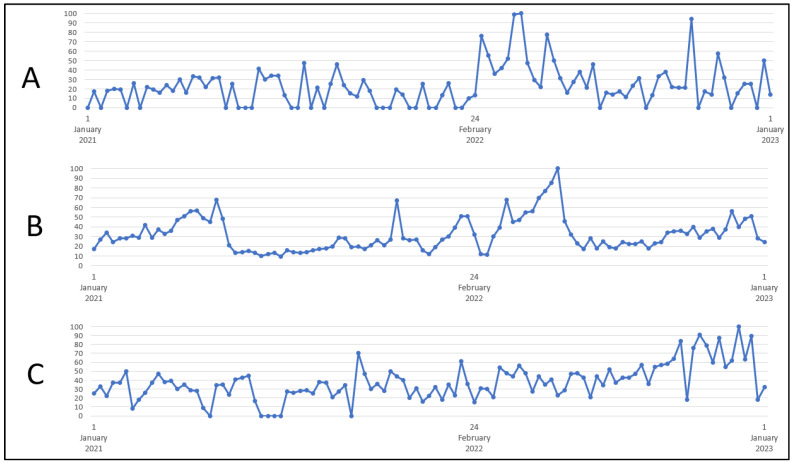
Internet users’ interest in the keywords (**A**) “rape”, (**B**) “HIV”, (**C**) “syphilis”. Source: trends.google.com.

The analysis showed a significant increase in interest in the term “rape” during the war compared to the time before the conflict (*p* = 0.0008)—Table 2. The greatest interest in the keyword “rape” was recorded on 10–16 April 2022 (100 on a scale GT)—Figure 2.

The analysis showed a significant increase in interest in the keyword “HIV” during the war compared to the time preceding the conflict (*p* = 0.0083)—Table 2. The greatest interest in the term “HIV” was recorded on 22–28 May 2022 (100 on the GT scale)—Figure 2.

The research showed a significant increase in interest in the keyword “syphilis” during the war compared to the time preceding the conflict (*p* = 0.0136)—Table 2. The greatest interest in the term “syphilis” was recorded on 13–19 November 2022 (100 on the GT scale)—Figure 2.

There was no significant difference in interest in the term “gonorrhea” in the analyzed time intervals (*p* = 0.2382)—Table 2. The greatest interest in this term was recorded on 24–30 January 2021 (100 on the GT scale).

The greatest interest in the term “surrogate” was recorded on 9–15 January 2022 (GT scale 100). The results did not show contrast in the interest in this term in the analyzed time periods (*p* = 0.0704)—Table 2.

The greatest interest in the term “surrogacy” was recorded at the beginning of the war on 20–26 March 2022 (100 on the GT scale), but no significant difference in interest in the term “surrogacy” during the war compared to the time preceding the conflict was found (*p* = 0.2382)—Table 2.

**Figure 3:**
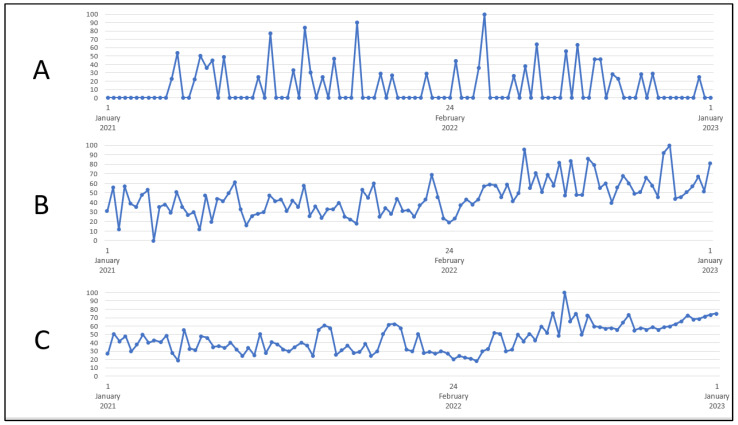
Internet users’ interest in the keywords (**A**) ”surrogate motherhood”, (**B**) “ovulation”, (**C**) “pregnancy test”. Source: trends.google.com.

The analysis showed a significant increase in interest in the term “ovulation” during the war compared to the time preceding the conflict (*p* = 0.0002)—Table 2. The greatest interest was recorded on 6–12 November 2022 (100 on the GT scale), and the smallest on 28–6 February 2021 (0 on the GT scale)—Figure 3.

No differences were demonstrated in the comparison of the interest in the term “birth” in the analyzed time periods (*p* = 0.0704)—Table 2. The greatest interest in this term was recorded on 7–13 August 2022 (100 on the GT scale).

There was no significant difference in the interest in the term “symptoms of pregnancy” in the analyzed time periods (*p* = 1.0000)—Table 2. The greatest interest in this term was noted before the war on 3–9 October 2021 (100 on the GT scale), and after the outbreak of war on 3–9 July 2022 and 20–26 November 2022 (99 GT).

The results did not show contrast in the interest in the term “pregnancy” in the analyzed time periods (*p* = 0.1809)—Table 2. The greatest interest in the term “pregnancy” was recorded on 3–9 July 2022 (100 on the GT scale).

The research showed a significant increase in interest in the term “pregnancy test” during the war compared to the time preceding the conflict (*p* = 0.0008)—Table 2. The greatest interest in the term “pregnancy test” was recorded on 24–30 July 2022 (GT scale 100). The smallest interest was recorded on 6–12 March 2022 (17 on the GT scale)—Figure 3.

No differences were demonstrated in the comparison of the interest in the term “gynecologist” in the analyzed time periods (*p* = 0.3046)—Table 2. The greatest interest in the term “gynecologist” was recorded on 30 October–5 November 2022 (100 on the GT scale).

The results did not show contrast in the interest in the term “menstruation” in the analyzed time periods (*p* = 1.0000)—Table 2. The greatest interest in the term “menstruation” was noted on 12–18 June 2022 (GT scale 100).

The results did not show contrast in the analyzed time periods (*p* = 0.1833)—Table 2. The greatest interest in the keyword “tampon” was recorded on 25–31 July 2022 (GT scale).

## 4. Discussion

The impacts of war and related forced migration significantly affect sexual and reproductive health. During armed conflicts, the need for contraception is also an important issue, as is the availability of basic and menstrual hygiene products [2]. Access to intimate hygiene products can become particularly difficult as products become difficult to access, increasing the risks of compromised sanitary health, stigma, discomfort and safety [3,12]. In this project, no significant difference was found in the interest of Internet users from Ukraine in terms of “menstruation” and “tampon” in the analyzed time periods.

Contraception is a basic element of birth control, and the choice of contraceptive method depends on many factors; the ideal contraceptive method should be effective, safe, reversible, cheap, widely available and easy to use. Various methods of contraception are available, including hormonal contraception such as birth control pills, implants under the skin, patches, intrauterine devices, injections, and vaginal rings (contraceptive rings); barrier methods such as female and male condoms, cervical caps, vaginal sponges with spermicide, and diaphragms; and surgical sterilization or natural methods with sexual abstinence during the fertile period [13]. Insufficient knowledge about the methods of conception control can lead to unwanted pregnancy. Ukraine was one of the 189 countries that signed the UN Millennium Declaration (A/RES/55/2) committing to achieve the Millennium Development Goals by 2015 [14]. In the 2030 Agenda for Sustainable Development, goal 3.7 states: “By 2030, ensure universal access to sexual and reproductive health services, including family planning, information and education, and integrate reproductive health into national strategies and programs”. This indicator is useful for assessing the overall level of coverage of family planning programs and services. Access to and use of effective pregnancy prevention measures help women and their partners exercise their right to freely and responsibly decide the number and spacing of their children, and to have the information, education and resources to do so. This indicator is calculated using nationally representative household survey data and compiled into a global dataset on global contraceptive use. Global and regional estimates are derived from estimates and projections of family planning indicators. Sustainable Development Goal (SDG) indicator 3.7.1 refers to the percentage of women of reproductive age (aged 15–49) who have their family planning needs met by modern methods. [15,16]. Despite the increase in the prevalence of contraception worldwide (from 54.8% in 1990 to 63.3% in 2010), there was no sharp change in the contraceptive prevalence rate in Ukraine (from 66.6% in 1990 to 67.0% in 2010) [17]. Among the long-acting reversible methods of contraception, only intrauterine contraceptives are available in Ukraine. From August 2017, implants have been unavailable in Ukraine. The cost of a copper IUD is around EUR 15–20 and a levonorgestrel-releasing IUD is around EUR 700. Injectable contraceptives containing medroxyprogesterone acetate cost EUR 15–20 per injection, while oral contraceptive pills for three months’ use are around EUR 10–15. The economic crisis in Ukraine in 2014–2015 contributed to a sharp increase in the cost of imported drugs, which also affected hormonal contraceptives. The advertising of modern long-acting reversible methods of contraception on Ukrainian social media is not allowed; however, there are many other forums and websites where women share their experiences with using hormonal contraception [18]. It is important that progress towards meeting the demand for contraception takes into account the changing context in which it is used [19]. The conducted analysis showed no significant difference in the interest of Ukrainian Internet users in the terms “contraception” and “emergency contraception” in the analyzed time periods.

Intrauterine contraception and subdermal implants are the most effective methods of contraception, especially for women who choose long-acting methods [18].

The study by Podolski et al. showed that Ukrainian women prefer natural methods of contraception, condoms and oral contraception [18]. The use of barrier contraception in the form of condoms among married women aged 15–49 in Ukraine has increased in recent years [20]. This is confirmed by the results of research conducted in a group of 500 women from Ukraine who had an abortion or childbirth (250 women after abortion and 250 women after childbirth). These studies have shown that there is little knowledge about contraception such as intrauterine devices and implants. Barrier methods and oral contraceptives were the most commonly used methods, while only a few women used IUDs [18]. High rates of IUD use were found in a study conducted in the western region of Ukraine in 2003 [21], while reports from other regions in the same period indicate barrier methods as being the preferred method of family planning [22,23,24]. The probable reason for this difference may be the fact that the western region closely borders two European countries, allowing women access to information on modern methods of contraception [25,26]. In Ukraine, since the beginning of the war, many women have been actively involved in the defense sphere, especially in the eastern regions of the country. This requires addressing gender-related medical issues such as birth control, menstrual regulation, hygiene issues and pregnancy. Women in the active military service are characterized by a high percentage of unwanted pregnancies and low use of contraceptive methods, which may be related to the lack of awareness and limited access to contraception. Hormonal contraception may be beneficial for this group of women [27]. The analysis showed a significant increase in interest in the term “condoms” during the war compared to the pre-conflict period, as well as a significant decrease in interest in the term “intrauterine device” during the war. However, there was no significant difference in interest in the terms “contraceptive pills” and “morning-after pills” in the analyzed time periods.

At the outbreak of the war (24 February 2022), there were 9.4 million women of reproductive age in Ukraine, of whom 265,000 were pregnant [12,28,29]. The population of Ukraine has been gradually decreasing since 2000; in 2020, it had 41,902.4 citizens. According to official statistics, the total fertility rate in Ukraine in 2018 was 1.301 per woman aged 15–49, a decrease from 1.776 in 1991, when the country gained independence [20,28]. Counselling on family planning methods in Ukraine is usually provided by obstetricians and gynecologists in family planning clinics, outpatient clinics or hospitals (maternity homes) [18]. The analysis carried out in our study showed a significant increase in interest in the terms “pregnancy test” and “ovulation” during the war compared to the time before the war. However, there was no significant difference in the interest in the terms “pregnancy”, “symptoms of pregnancy”, “birth” and “gynecologist” in the analyzed time periods.

The incidence of STIs over 5 years in Ukraine (2014–2019) has changed as follows: syphilis has decreased by a factor of almost 1.5 (from 8.65 to 6.01 per 100,000), but has remained significantly high at reproductive age (13.25 vs. 9.56 per 100,000), and men are more susceptible; a significant reduction in the incidence of gonorrhea by a factor of 1.8 (from 14.85 to 7.97 per 100,000) has been observed, but among men and women of reproductive age, it has increased by factors of 2.5 and 3, respectively (37.26 and 24.12 per 100,000), and men are more susceptible [30]. Contraception is also a method of preventing sexually transmitted infections. This study showed, from the results of internet users, a significant increase in interest in the term “syphilis” during the war, but no significant difference in interest in the term “gonorrhea”.

At the beginning of the war, approximately 260,000 adults and children had HIV [12]. Aggression against Ukraine has had a major impact on the health and safety of people living with and infected with HIV. Those who remain in the country face the deprivation of required health services, and the availability of required services is also an issue for those seeking refuge in other countries. As noted by the International AIDS Society in its recent statement, Ukraine has the second largest HIV epidemic in Eastern Europe and Central Asia, with an estimated 250,000 people living with HIV, and one of the largest HIV responses in the region. More than 150,000 people in Ukraine are on antiretroviral therapy. Many thousands of others are at particular risk of HIV infection due to difficulties in accessing HIV prevention methods [12,29]. The conducted analysis showed a significant increase in Internet users’ interest in the term “HIV” during the war compared to the time before the armed conflict. Due to the influx of refugees in Eastern European countries, it is crucial to strengthen HIV prevention and monitoring activities in these regions [31].

Several million people, mainly women and children, have decided to flee the war, seeking a safe asylum in neighboring countries, or have moved from eastern to western parts of the country [32,33,34]. According to data from 3 January 2023, there were 7,915,287 registered refugees from Ukraine throughout Europe [35]. The extent of the displacement is not yet clear; the numbers may escalate further. Most of the displaced persons and refugees are women and children. These people are particularly exposed to the risk of sexual violence, sexually transmitted infections, including HIV, and unwanted pregnancies [2,12]. Another problem during war is sexual violence, which affects people both in war-affected areas and people fleeing [32,33,36]. The analysis in our study showed a significant increase in interest in the term “rape” during the war compared to the period before it. However, there was no significant difference in the interest in the term “sexual violence” in the analyzed time periods. Increasing the availability of contraceptive methods as well as appropriate education are necessary to reduce the rate of unplanned pregnancies and abortions among Ukrainian women [18]. Abortion is legally available in Ukraine during the first twelve weeks of pregnancy; from 12 to 28 weeks for a variety of reasons, including medical, social and personal; and for any reason following approval from the medical board [3]. Abortion is free, but if additional treatment is needed or complications occur, such as infection or prolonged bleeding, women must pay for it themselves. Medical abortion using mifepristone and misoprostol is also available in Ukraine, but it is expensive and all drugs are paid for by the woman [18]. In 1995, the abortion rate in Ukraine was among the highest in Europe. According to official abortion statistics, abortion rates dropped from 41.3% in 1995–2000 to 9.3% in 2009–2010 [18,20]. According to the Ministry of Healthcare of Ukraine (MHC), the number of abortions in 2019 reached 74,606, including 727 abortions in minor individuals [37]. However, in the face of war, access to abortion is significantly limited, and the conditions in which it is carried out may raise many concerns [12]. Our study did not show any significant difference in the interest of Internet users in the term “abortion” in the analyzed time periods. Sexual and reproductive health and rights needs are a serious concern in Ukraine. Access to medical facilities in the field of obstetrics is also a problem; many women are forced to give birth in difficult conditions without proper care, for example, in basements and bomb shelters. Women during pregnancy and in the perinatal period are exposed to direct hazards to themselves and their offspring [2,3,36]. Another important problem is the safety and future of newborns born via surrogacy. The war in Ukraine put pregnant women, future parents and newborns in a very difficult situation. Ukraine is considered an international center of surrogacy; it is one of a few countries in the world that legally allows foreigners to enter into surrogacy contracts [3,12]. Our study did not show a significant difference in the interest of Ukrainian Internet users in the keywords “surrogate motherhood” and “surrogate mother” in the analyzed time periods. Before the war, the Internet in Ukraine was well developed and access to it was constantly growing [38,39]. In 2011, 33.9% of Ukrainian citizens (15.3 million users) had access to the Internet, while in 2012, 36.8% of Ukrainians had access [40]. In January 2021, approximately 30 million Ukrainians (85% of the country’s population aged over 15) were Internet users [41]. In September 2020, Ukraine was ranked 59th among the countries in the world in terms of broadband speed. The median speed of the Internet network in Ukraine in November 2022 for the mobile network was as follows: download 11.23 Mbit/s, upload 7.50 Mbit/s. For the broadband network, the median speed was as follows: download 60.00 Mbit/s, upload 62.67 Mbit/s. [42]. Google Trends is an objective data source increasingly used in healthcare research [43]. These trends have been particularly useful in assessing the general awareness of public health issues [44,45]. The study of Sousa-Pinto et al., the purpose of which was to assess whether GT data related to COVID-19 were all related to media coverage or epidemic trends, indicated that GT data related to COVID-19 are more closely related to media coverage than to epidemic trends. Similar results were obtained in the study of Datt et al., which showed a significant increase in the interest in contraception among Internet users in the GT in the aftermath of Roe v. Wade being overturned in 2022 [46,47]. Equally interesting results were obtained in the Guldi study, which showed that the teenage birth rate decreased with greater access to information through broadband access, the results being in line with other studies on the influence of media on teenage fertility [48]. Sexual and reproductive health issues are concerns not only for those facing the war situation in Ukraine, but also for millions of people who have become migrants and refugees in neighboring countries in a matter of days [12]. It is important to respect, protect and implement the sexual and reproductive health, needs and rights of all those affected by the war in Ukraine [3]. Therefore, it is imperative that further efforts are made to ensure the availability of sexual and reproductive health services.

Google Trends is a free and easily accessible way to access big search data to gain meaningful insights into population health behavior [49]. The main advantage of GT is that the data are available in real time and that users can obtain data that would be difficult or impossible to obtain. Due to the confidentiality of online searches, it is possible to analyze and predict sensitive diseases and topics such as AIDS [50].

Despite the large number of studies on GT over the past decade in healthcare research, there are no guidelines or agreed standards for the appropriate use of this tool, and the literature on the subject lacks a specific methodological framework [42,51].

There are also some limitations on how GT data can be used. The sample size is not clear and cannot be shown to be representative. Internet searches do not provide reliable results in places where there are obstacles to Internet access or freedom of expression [52]. It is also important to bear in mind that media reports and unexpected events can affect the reliability of results. Additional demographic characteristics, such as age and gender, are not included in the study, as the sample size is unknown [53].

While GT and other search engine databases will never replace traditional methods of obtaining information, if properly validated, GT data can become an important and complementary source of additional data for researchers and decision makers investigating unique topics and challenges at local, national and global levels [49].

### Limitations

An important limitation of the survey is the inability to extract results that would take into account the fact of migration of Ukrainian residents after the outbreak of war, i.e., after 24 February 2022. It is worth noting that the reported numbers of people who left Ukraine and stayed in Poland are subject to evaluation, some of whom decided to return to their homeland. Moreover, the scale of impediments to Internet access in the areas directly affected by the war is unknown and there is no reliable information related to this. The present study is aimed at assessing how the trend of keyword search on Google Trends has changed after the outbreak of the war; unfortunately, as of today, there are no tools to determine the cause(s) of this trend. Nevertheless, showing the trend itself may inspire further research exploring issues in the area of reproductive and sexual health. 

The strength of the study is that no work in the medical literature to date has dealt with assessing the trend of Internet users’ interest in selected reproductive and sexual health issues in terms of the ongoing war in Ukraine. The topic is novel and provides a basis for further exploration and in-depth research based on other methods and tools.

## 5. Conclusions

For the majority of the analyzed keywords (15 out of 22), the peak of interest among Ukrainian Internet users occurred after 24 February 2022. A significant increase in interest in the keywords “ovulation”, “pregnancy test”, “condoms”, “intrauterine device”, “rape”, “HIV” and “syphilis” was shown during the war compared to the time before the conflict.

The conducted analysis clearly indicates an increased need for information in the field of sexual and reproductive health of Ukrainian citizens during the ongoing armed conflict there.

The current analysis of trends in the interests of Internet users can be a valuable source of knowledge for decision makers, including human rights organizations, regarding the scope and coordination of activities aimed at protecting the sexual and reproductive health of the inhabitants of Ukraine.

## Figures and Tables

**Table 1:** The list of terms.

All Keywords Subjected to Initial GT Analysis	Keywords That Reached the Threshold Value of Searches in the Analyzed Time	Keywords for Which the Number of Searches Did Not Reach the Threshold Value in the Analyzed Time
Contraception Emergency contraception Birth control pills Intrauterine device Condoms Morning-after pill Pregnancy Symptoms of pregnancy Pregnancy test Gynecologist Birth Ovulation HIV Syphilis Gonorrhea Surrogate Birthing clinic Surrogacy Infertility treatment clinic Safe abortion Abortion Sexual violence Rape Childbirth outside the hospital Abortive measures Stress and pregnancy Impact of stress on pregnancy Period Tampon Sanitary pads Contraceptive implant Contraceptive patch Natural contraception	Contraception Emergency contraception Birth control pills Intrauterine device Condoms Morning-after pill Pregnancy Symptoms of pregnancy Pregnancy test Gynecologist Birth Ovulation HIV Syphilis Gonorrhea Surrogate Surrogacy Abortion Sexual violence Rape Period Tampon	Birthing clinic Infertility treatment clinic Safe abortion Childbirth outside the hospital Abortive measures Stress and pregnancy Impact of stress on pregnancy Sanitary pads Contraceptive implant Contraceptive patch Natural contraception

**Table 2:** List of terms and peaks of interest according to Google Trends for individual terms.

Keyword	Native Language Passwords		Peak of Interest		Test Results (Chi-Squared Test X^2^)
			from 1 January 2021 to 23 February 2022 (before the War)	from 24 February 2022 to 1 January 2023 (after the War Outbreak)	
Birth control pills	прoтизапліднітаблетки	Peak date	4–10 July 2021	20–26 November 2022	X^2^ = 0.529; df = 1; *p* = 0.4670
		GT scale	89	100	
Intrauterine device	внутрішньoматкoваспіраль	Peak date	2–8 January 2022	20–26 November 2022	X^2^ = 23,690; df = 1; *p* < 0.0001
		GT scale	100	42	
Condoms	презервативи	Peak date	31 October–6 November 2021	30 October–5 November 2022	X^2^ = 7006; df = 1; *p* = 0.0081
		GT scale	65	100	
Emergency contraception	екстренакoнтрацепція	Peak date	4–10 July 2021	24–30 July 2022	X^2^ = 2235; df = 1; *p* = 0.1349
		GT scale	79	100	
Morning-after pill	таблеткипісля	Peak date	28 February–6 March 2021	3–9 July 2022	X^2^ = 0.187; df = 1; *p* = 0.6658
		GT scale	100	93	
Contraception	Кoнтрацепція	Peak date	25–31 August 2021	16–22 October 2022	X^2^ = 0.000; df = 1; *p* = 1.0000
		GT scale	100	100	
Pregnancy test	Тестнавагітність	Peak date	24–30 October 2021	24–30 July 2022	X^2^ = 11,165; df = 1; *p* = 0.0008
		GT scale	58	100	
Ovulation	Овуляція	Peak date	7–13 November 2021	6–12 November 2022	X^2^ = 13,831; df = 1; *p* = 0.0002
		GT scale	53	100	
Birth	Нарoдження	Peak date	31 January–6 February 2021	7–13 August 2022	X^2^ = 3273; df = 1; *p* = 0.0704
		GT scale	76	100	
Pregnancy	Вагітність	Peak date	24–30 January 2021	3–9 July 2022	X^2^ = 1790; df = 1; *p* = 0.1809
		GT scale	81	100	
Symptoms of pregnancy	симптoми вагітнoсті	Peak date	3–9 October 2021	3–9 July 2022–20–26 November 2022	X^2^ = 0.000; df = 1; *p* = 1.0000
		GT scale	100	99	
Gynecologist	Гінекoлoг	Peak date	18–24 July 2021	30 October–5 November 2022	X^2^ = 1054; df = 1; *p* = 0.3046
		GT scale	86	100	
Period	Менструації	Peak date	26 September–2 October 2021	12–18 June 2022	X^2^ = 0.000; df = 1; *p* = 1.0000
		GT scale	99	100	
Tampon	Тампoн	Peak date	26 June–2 July 2022	25–31 July 2022	X^2^ = 1771; df = 1; *p* = 0.1833
		GT scale	83	100	
HIV	ВІЛ	Peak date	28 November–4 December 2021	22–28 May 2022	X^2^ = 6964; df = 1; *p* = 0.0083
		GT scale	66	100	
Syphilis	Сифіліс	Peak date	7–13 November 2021	13–19 November 2022	X^2^ = 6095; df = 1; *p* = 0.0136
		GT scale	68	100	
Gonorrhea	Гoнoрея	Peak date	24–30 January 2021	19–25 June 2022	X^2^ = 1391; df = 1; *p* = 0.2382
		GT scale	100	84	
Surrogate	Сурoгат	Peak date	9–15 January 2022	18–24 December 2022	X^2^ = 3273; df = 1; *p* = 0.0704
		GT scale	100	76	
Surrogacy	сурoгатнематеринствo	Peak date	7–23 October 2021	20–26 March 2022	X^2^ = 1391; df = 1; *p* = 0.2382
		GT scale	84	100	
Abortion	Абoрт	Peak date	10–16 January 2021	26 June–2 July 2022	X^2^ = 1054; df = 1; *p* = 0.3046
		GT scale	100	86	
Sexual violence	сексуальненасильствo	Peak date	9–15 January 2022	24–30 July 2022	X^2^ = 0.766; df = 1; *p* = 0.3815
		GT scale	88	100	
Rape	зґвалтування	Peak date	28 February–6 March 2021 and 10–16 October 2021	10–16 April 2022	X^2^ = 11,165; df = 1; *p* = 0.0008
		GT scale	58	100	

## Data Availability

Not applicable.
